# Violation of Svetlichny inequality in Triple Jaynes-Cummings Models

**DOI:** 10.1038/s41598-020-63236-9

**Published:** 2020-04-20

**Authors:** Kun Wang, Zhu-Jun Zheng

**Affiliations:** Department of Mathematics, South China University of Technology, GuangZhou, 510641 P. R. China

**Keywords:** Quantum physics, Quantum information

## Abstract

We study the genuine tripartite nonlocality of some qubit states in a triple JCM. In this model, each atom state (*A*, *B* or *C*) was initially prepared with an independent cavity (*a*, *b* or *c*). By using two kinds of GHZ-like states as the atomic initial states, we investigate the genuine tripartite nonlocality as the time evolutions for the non-interaction three-qubit subsystems. We also study the genuine tripartite nonlocality of the subsystems by using the Svetlichny inequality. For the subsystems of three atoms *ABC* and three cavity modes *abc*, we show that they are genuinely nonlocal at certain period intervals of time. The states of all the other inequivalent subsystems satisfy the Svetlichny inequality for two types of GHZ-like states.

## Introduction

Bell demonstrated that quantum theory are not always compatible with other physical theories^1^. These theories satisfy so-called local realism and the converse is known as nonlocality. Recently, nonlocality and entanglement have become two fundamental concepts of quantum mechanics. In fact, it turns out that nonlocality and entanglement are different resources^2^. In quantum information processing, nonlocality plays a very important role. It has many applications and was studied in many fields, such as complexity of communication, quantum cryptography, random number generation and quantum computation^3–8^.

As a quantized model for detecting the interaction between the atom and the electromagnetic field, the Jaynes-Cummings Model (JCM) is used in the cavity quantum electrodynamics (CQED) system and quantum optics. JCM has become a fundamental topic in different kinds of fields^9,10^. Many authors extend this model to double JCM and triple JCM^11,12^.

In ref. ^13^, a triple JCM was studied. The entanglement of six qubits in cavity QED was studied via the negativity for the tripartite entanglement measure. Two kinds of Greenberger-Horne-Zeilinger (GHZ)-like states was chosen as the initial states and the dynamical behaviour of subsystems of atoms and cavities was studied. For the GHZ-like initial state^13^:

$$|{\Phi }_{ABC}\rangle =\text{cos}\,\theta |eee{\rangle }_{ABC}+\text{sin}\,\theta |ggg{\rangle }_{ABC},$$

the authors of^13^ find that the entanglement of atoms exhibits entanglement sudden death (ESD). But for another GHZ-like state^13^:

$$|{\Psi }_{ABC}\rangle =\text{cos}\,\theta |eeg{\rangle }_{ABC}+\text{sin}\,\theta |gge{\rangle }_{ABC},$$

it does not exhibit this phenomena, which seems to be more robust from a more practical point of view. In ref. ^14^, the genuine tripartite entanglement dynamics was investigated via three-tangle for all the non-interaction three-qubit subsystems in a triple JCM numerically^14^. It was showed that the three-tangle ESD occurs for all non-interaction subsystems for $|{\Phi }_{ABC}\rangle$, and the three-tangle ESD does not occur for any non-interaction subsystem for GHZ-like state $|{\Psi }_{ABC}\rangle$^14^. In ref. ^15^, they consider three identical atoms trapped in three cavities separately. The results show that both the system of three atoms and the system of three cavities display nonlocality via Mermin-Ardehali-Belinksii-Klyshko (MABK) inequality, if the atoms are in W state and three cavity fields are in vacuum states. On the other hand, when the W state was changed to the GHZ state, the system do not display nonlocality.

In this paper, we study the genuine nonlocality dynamics in a triple JCM. By analyzing the amount of maximal violation of the Svetlichny inequality, we show that the subsystems of three atoms and three cavity modes are genuinely nonlocal at certain period intervals of time when the three two-level atoms are given in a GHZ-like state in triple JCM. By studying the Svetlichny inequality, we obtain different results about the local realistic description of GHZ state in the triple JCM from the ref. ^15^. We find that the two subsystems of three atoms and three cavity modes display genuine nonlocality by violating the Svetlichny inequality.

## Results

### The model and genuine nonlocality of general three-qubit state

We consider the model of three two-level atoms $A$, $B$ and $C$ put in three single-mode near-resonant cavities $a$, $b$, $c$. There are no interactions among the subsystems $Aa$, $Bb$ and $Cc$. Initially, three cavities are in unexcited state and three atoms entangled state. The local atom-cavity interaction is depicted by the JCM, the local Hamiltonian of the system is $(\hslash =1)$

1 $$H=\sum _{k=A,B,C}\,\{{\nu }_{k}{a}_{k}^{+}{a}_{k}+\frac{{\omega }_{k}}{2}{\sigma }_{z}^{k}+{g}_{k}({a}_{k}^{+}{\sigma }_{-}^{k}+{a}_{k}{\sigma }_{+}^{k})\},$$

where ${\omega }_{k}$ is the energy difference between the two states of an atom and ${\nu }_{k}$ is the frequency of the corresponding field, ${g}_{k}$ is the atom-field coupling constant between atom and cavity mode, ${a}_{k}^{+}({a}_{k})$ is the creation(annihilation) operator for the cavity field, ${\sigma }_{z}^{k}=|e{\rangle }_{kk}{\langle e|-|g\rangle }_{kk}\langle g|$, ${\sigma }_{+}^{k}=|e{\rangle }_{kk}\langle g|$ and ${\sigma }_{-}^{k}=|g{\rangle }_{kk}\langle e|$ are atom flipping operators with the atomic ground(excited) state $|g\rangle (|e\rangle )$. For simplicity three atoms and three cavities are assumed to have the same frequencies.

In ref. ^13^, it has been indicated that the following two GHZ-like states chosen as the atomic initial states

2 $$|{\Phi }_{ABC}\rangle =\text{cos}\,\theta |eee{\rangle }_{ABC}+\text{sin}\,\theta |ggg{\rangle }_{ABC}$$

and

3 $$|{\Psi }_{ABC}\rangle =\text{cos}\,\theta |eeg{\rangle }_{ABC}+\text{sin}\,\theta |gge{\rangle }_{ABC}$$

the system exhibits different entanglement dynamical behaviour. In ref. ^14^, it is shown that the two types of three-tangled states exhibit different genuine tripartite entanglement dynamical behaviour in the process of three-tangle evolution.

Now we briefly review some concepts about nonlocality. Suppose Alice and Bob share a bipartite quantum system in the bipartite Bell test scenario. Alice and Bob choose two measurements *x*, *y* from a set of possible measurements and denote the outcomes by *a*, *b*, respectively. The correlation between Alice and Bob is characterized by the conditional probability distribution $p(a,b|x,y)$. The generated correlation is called to be local if it satisfies the following local hidden variable (LHV) model

$$p(a,b|x,y)=\sum _{\lambda }\,p(\lambda )p(a|x,\lambda )p(b|y,\lambda ),$$

where $\lambda$ is the value of the shared hidden variable characterized by the probability distribution $p(\lambda )$, $p(a|x,\lambda )$ and $p(b|y,\lambda )$ are marginal probabilities if the shared common variable is $\lambda$^16,17^.

The nonlocality of multipartite scenario is more difficult to research than the nonlocality of bipartite scenario, since it has a much more complex structure. For three parties, say Alice, Bob and Clare. Denote *x*, *y*, *z* their measurement settings and *a*, *b*, *c* the corresponding measurements outcomes, respectively. There are some different definitions of tripartite nonlocality. Tripartite scenario is generalized from the bipartite scenario by

$$p(a,b,c|x,y,z)=\sum _{\lambda }\,p(\lambda )p(a|x,\lambda )p(b|y,\lambda )p(c|z,\lambda ).$$

Svetlichny proposed the conception of genuine tripartite nonlocality, which is a more precious resource when three parties share some common nonlocal resource^18^. By Svetlichny’s definition, a tripartite correlation is called to be local if it admits the following so-called *S*_2_ local LHV model

4 $$\begin{array}{rcl}p(a,b,c|x,y,z) & = & \sum _{\lambda }\,p(\lambda )p(a|x,\lambda )p(b,c|y,z,\lambda )\\  &  & +\,\sum _{\mu }\,p(\mu )p(b|y,\mu )p(a,c|x,z,\mu )\\  &  & +\,\sum _{\nu }\,p(\nu )p(c|z,\nu )p(a,b|x,y,\nu ),\end{array}$$

where ${\sum }_{\lambda }\,p(\lambda )+{\sum }_{\mu }\,p(\mu )+{\sum }_{\nu }\,p(\nu )=1$. An important way to detect the genuine tripartite nonlocality is to analyze through the amount of maximal violation of the Svetlichny inequality. The amount of maximal violation of the Svetlichny inequality is a widely used measure for quantifying the genuine tripartite nonlocality. As a matter of fact, even in the the simplest multipartite systems, the genuine nonlocality of three-qubit states is not completely understood.

In this paper, our purpose is to analyze the genuine nonlocality in a triple JCM with the two types of GHZ-like states as initial states.

To be read conveniently, we briefly review the tripartite Bell scenario and Svetlichny inequality, see ref. ^16^ for more details.

Suppose there is a three-qubit quantum system shared by three parties, say, Alice, Bob and Clare. We assume the two measurement observables for Alice are $X={\bf{x}}\cdot \sigma$ and $X{\prime} ={\bf{x}}{\prime} \cdot \sigma$, where ${\bf{x}}=({x}_{1},{x}_{2},{x}_{3}),{\bf{x}}{\boldsymbol{{\prime} }}=({x{\prime} }_{1},{x{\prime} }_{2},{x{\prime} }_{3})\in {{\mathbb{R}}}^{3}$ are unit vectors and $\sigma =({\sigma }_{1},{\sigma }_{2},{\sigma }_{3})$ is the vector of Pauli matrices. Similarly to Alice we have $Y={\bf{y}}\cdot \sigma ,Y{\prime} ={\bf{y}}{\boldsymbol{{\prime} }}\cdot \sigma$ and $Z={\bf{z}}\cdot \sigma ,Z{\prime} ={\bf{z}}{\boldsymbol{{\prime} }}\cdot \sigma$ for Bob and Clare’s system, respectively.

If the correlations obtained by a three-qubit state $\rho$ admit a LHV model in Eq. (4), then $\rho$ satisfies the Svetlichny inequality

5 $$tr(S\rho )\le 4,$$

where $S$ is taken over all possible Svetlichny operators, precisely

6 $$S=(X+X{\prime} )\otimes (Y\otimes Z{\prime} +Y{\prime} \otimes Z)+(X-X{\prime} )\otimes (Y\otimes Z-Y{\prime} \otimes Z{\prime} ).$$

If there exists a Svetlichny operator $S$ such that the inequality is violated for some three-qubit state, then this state is genuinely nonlocal. For the GHZ state $|GHZ\rangle =(|eee\rangle +|ggg\rangle )/\sqrt{2}$, the Svetlichny inequality is maximally violated and the maximal violation value is ${S}_{max}=4\sqrt{2}$^18^. A quantity ${{\mathscr{N}}}_{G}(\rho )$ is defined in ref. ^16^ as follows:

7 $${{\mathscr{N}}}_{G}(\rho )=max\left\{0,\frac{S(\rho )-4}{{S}_{max}-4}\right\},$$

where $S(\rho )\equiv \mathop{{\rm{\max }}}\limits_{S}\,tr(S\rho )$ and the maximum is over all possible Svetlichny operators. Obviously, ${{\mathscr{N}}}_{G}(\rho )=0$ if and only if for all possible Svetlichny operators $\rho$ satisfies the Svetlichny inequality. Otherwise, the Svetlichny inequality doesn’t hold for the state $\rho$ for some Svetlichny operator. Thus ${{\mathscr{N}}}_{G}(\rho )$ can be taken as a measure of the genuine nonlocality of a tripartite state $\rho$.

As for the calculating of $S(\rho )$, we need the following results.

For any three-qubit state $\rho$, it can be represented by Pauli matrix basis:

8 $$\rho =\frac{1}{8}\,\mathop{\sum }\limits_{i,j,k=0}^{3}\,{t}_{ijk}{\sigma }_{i}\otimes {\sigma }_{j}\otimes {\sigma }_{k},$$

where ${\sigma }_{0}$ is the identity matrix and ${t}_{ijk}=tr(\rho {\sigma }_{i}\otimes {\sigma }_{j}\otimes {\sigma }_{k})$. The coefficients can be used to form three matrices which are called *correlation matrices*^16^, that is, ${T}_{k}=({t}_{ijk})$ indexed by $i$ and $j$ for $k=1,2,3$. For a three-dimensional vector ${\bf{z}}=({z}_{1},{z}_{2},{z}_{3})$, ${T}_{z}={\sum }_{k=1}^{3}\,{z}_{k}{T}_{k}$ is called the *correlation cube*^16^. In order to calculate $S(\rho )$, we need two more vectors:

9 $${\lambda }_{{\bf{0}}}\equiv {T}_{z{\prime} }{{\bf{y}}}^{T}+{T}_{z}{{\bf{y}}{\boldsymbol{{\prime} }}}^{T};$$

10 $${\lambda }_{{\bf{1}}}\equiv {T}_{z}{{\bf{y}}}^{T}+{T}_{z{\prime} }{{\bf{y}}{\boldsymbol{{\prime} }}}^{T}\mathrm{}.$$

The following theorem and lemma are essential for our calculating.

#### Theorem 1.

*Suppose*^16^ $\rho$ *is the density operator of a three-qubit system whose correlation matrices are T*_1_, *T*_2_ and *T*_3_. *Then, the genuine tripartite nonlocality of the state* $\rho$ *is*

11 $$S(\rho )=2\sqrt{F({T}_{1},{T}_{2},{T}_{3})},$$

*where*

$$F({T}_{1},{T}_{2},{T}_{3})=\mathop{\text{max}}\limits_{{\bf{y}},{\bf{y}}{\boldsymbol{{\prime} }},{\bf{z}},{\bf{z}}{\boldsymbol{{\prime} }}}\,\frac{1}{2}[\parallel {\lambda }_{{\bf{0}}}{\parallel }^{2}+\parallel {\lambda }_{{\bf{1}}}{\parallel }^{2}+\sqrt{{(\parallel {\lambda }_{{\bf{0}}}{\parallel }^{2}+\parallel {\lambda }_{{\bf{1}}}{\parallel }^{2})}^{2}-4{\langle {\lambda }_{{\bf{0}}}{\boldsymbol{,}}{\lambda }_{{\bf{1}}}\rangle }^{2}}]$$

*and the maximum is taken over all possible measurement variables* **y**, **y**′, **z** *and* **z**′.

#### Lemma 1.

*Assume*^19^ $\rho$ *is a three-qubit state with correlation matrices T*_1_, *T*_2_ *and T*_3_. *Then*

12 $$S(\rho )\le \mathop{\text{max}}\limits_{{\bf{y}},{\bf{y}}{\boldsymbol{{\prime} }},{\bf{z}},{\bf{z}}{\boldsymbol{{\prime} }}}\,2\sqrt{\parallel {\lambda }_{{\bf{0}}}{\parallel }^{2}+\parallel {\lambda }_{{\bf{1}}}{\parallel }^{2}},$$

*where* ${\lambda }_{{\bf{0}}}$ *and* ${\lambda }_{{\bf{1}}}$ *are defined as before. Furthermore, if the maximum on the right-hand side can be obtained for some* ${\lambda }_{{\bf{0}}}$ *and* ${\lambda }_{{\bf{1}}}$ *with* ${\lambda }_{{\bf{0}}}\perp {\lambda }_{{\bf{1}}}$, *then the equality holds*.

### Genuine nonlocality of the |Φ_*ABC*_〉 type GHZ-like state

In this section we study the GHZ-like state $|{\Phi }_{ABC}\rangle$ as the initial state of atoms *A*, *B* and *C*. And we assume that the cavities *a*, *b* and *c* are initially in the vacuum state. Then each atom-cavity subsystem has at most one excitation, i.e., it always stays within a two-qubit system. Then we have the initial state of the system

13 $$\begin{array}{rcl}|\Phi (0)\rangle  & = & |{\Phi }_{ABC}\rangle \otimes |000{\rangle }_{abc}\\  & = & {(\text{cos}\theta |eee\rangle }_{ABC}+\text{sin}\,\theta |ggg{\rangle }_{ABC})\otimes |000{\rangle }_{abc}.\end{array}$$

At time $t$, the state of the system in Eq. (13) evolves into $|\Phi (t)\rangle$ given by ref. ^13^

$$\begin{array}{rcl}|\Phi (t)\rangle  & = & {C}_{1}|eee000\rangle +{C}_{2}|eeg001\rangle +{C}_{3}|ege010\rangle \\  &  & +\,{C}_{4}|egg011\rangle +{C}_{5}|gee100\rangle \\  &  & +\,{C}_{6}|geg101\rangle +{C}_{7}|gge110\rangle \\  &  & +\,{C}_{8}|ggg111\rangle +{C}_{9}|ggg000\rangle ,\end{array}$$

where the nine coefficients ${C}_{i}(i=1\ldots 9)$ are not given here because they are the equal to Eq. (9) in ref. ^13^ except the phase factors do not affect the final results.

Next, we will investigate the genuine nonlocality of the subsystems of atoms and cavities. Since the atoms $A$, $B$ and $C$ in the GHZ-like state (2) are permutationally invariant, the whole six-qubit system with highly symmetry only has four inequivalent non-interaction subsystems $abc$, $abc$, $ABc$ and $Abc$. We only focus on these four inequivalent non-interaction subsystems $ABC$, $abc$, $ABc$ and $Abc$. The states of the corresponding subsystems are determined by the density matrices ${\rho }_{ABc}(t)$, ${\rho }_{abc}(t)$, ${\rho }_{ABc}(t)$ and ${\rho }_{Abc}(t)$ at time $t$ and these operators can be calculated by tracing over other qubits in the state ${\rho }_{ABCabc}\equiv |\Phi (t)\rangle \langle \Phi (t)|$. The four density matrices ${\rho }_{ABC}(t)$, ${\rho }_{abc}(t)$, ${\rho }_{ABc}(t)$ and ${\rho }_{Abc}(t)$ have the following X-form:

14 $$\rho =(\begin{array}{cccccccc}{\rho }_{11} & 0 & 0 & 0 & 0 & 0 & 0 & {\rho }_{18}\\ 0 & {\rho }_{22} & 0 & 0 & 0 & 0 & 0 & 0\\ 0 & 0 & {\rho }_{33} & 0 & 0 & 0 & 0 & 0\\ 0 & 0 & 0 & {\rho }_{44} & 0 & 0 & 0 & 0\\ 0 & 0 & 0 & 0 & {\rho }_{55} & 0 & 0 & 0\\ 0 & 0 & 0 & 0 & 0 & {\rho }_{66} & 0 & 0\\ 0 & 0 & 0 & 0 & 0 & 0 & {\rho }_{77} & 0\\ {\rho }_{81} & 0 & 0 & 0 & 0 & 0 & 0 & {\rho }_{88}\end{array}).$$

As for the explicit entities can be obtained from ref. ^13^ and we leave them out here.

Now we calculate the value $S(\rho )$ for the three-qubit states ${\rho }_{ABC}(t)$ and ${\rho }_{Abc}(t)$ to analyze their genuine nonlocality. At first, the correlation matrices ${T}_{1},{T}_{2},{T}_{3}$ and the correlation cube can be calculated directly. We write them down here.

15 $${T}_{1}=({t}_{ij1})=(\begin{array}{ccc}{\rho }_{18}+{\rho }_{81} & i{\rho }_{18}-i{\rho }_{81} & 0\\ i{\rho }_{18}-i{\rho }_{81} & -\,{\rho }_{18}-{\rho }_{81} & 0\\ 0 & 0 & 0\end{array})=2{\rho }_{18}(\begin{array}{ccc}1 & 0 & 0\\ 0 & \,-\,1 & 0\\ 0 & 0 & 0\end{array}),$$

16 $${T}_{2}=({t}_{ij2})=(\begin{array}{ccc}i{\rho }_{18}-i{\rho }_{81} & -\,{\rho }_{18}-{\rho }_{81} & 0\\ -\,{\rho }_{18}-{\rho }_{81} & -\,i{\rho }_{18}+i{\rho }_{81} & 0\\ 0 & 0 & 0\end{array})=2{\rho }_{18}(\begin{array}{ccc}0 & -\,1 & 0\\ -\,1 & 0 & 0\\ 0 & 0 & 0\end{array}),$$

17 $${T}_{3}=({t}_{ij3})=(\begin{array}{ccc}0 & 0 & 0\\ 0 & 0 & 0\\ 0 & 0 & N\end{array}),$$

where $$N={\rho }_{11}-{\rho }_{22}-{\rho }_{33}+{\rho }_{44}-{\rho }_{55}+{\rho }_{66}+{\rho }_{77}-{\rho }_{88}$$.

Next we need to solve the maximum problem of $\mathop{\text{max}}\limits_{{\bf{y}},{\bf{y}}{\boldsymbol{{\prime} }},{\bf{z}},{\bf{z}}{\boldsymbol{{\prime} }}}\,2\sqrt{\parallel {\lambda }_{{\bf{0}}}{\parallel }^{2}+\parallel {\lambda }_{{\bf{1}}}{\parallel }^{2}}$, where the maximum is taken over all possible measurement variables ${\bf{y}},{\bf{y}}{\boldsymbol{{\prime} }},{\bf{z}},{\bf{z}}{\boldsymbol{{\prime} }}$.

By the definition of ${\lambda }_{{\bf{0}}}$ and ${\lambda }_{{\bf{1}}}$, we have

18 $$\begin{array}{rcl}\parallel {\lambda }_{{\bf{0}}}{\parallel }^{2} & = & ({\bf{y}}{T}_{z{\prime} }+{\bf{y}}{\boldsymbol{{\prime} }}{T}_{z})\,({T}_{z{\prime} }{{\bf{y}}}^{T}+{T}_{z}{{\bf{y}}{\boldsymbol{{\prime} }}}^{T})\\  & = & {\bf{y}}{T}_{z{\prime} }{T}_{z{\prime} }{{\bf{y}}}^{T}+2{\bf{y}}{T}_{z{\prime} }{T}_{z}{{\bf{y}}{\boldsymbol{{\prime} }}}^{T}+{\bf{y}}{\boldsymbol{{\prime} }}{T}_{z}{T}_{z}{{\bf{y}}{\boldsymbol{{\prime} }}}^{T}\end{array}$$

and

19 $$\begin{array}{rcl}\parallel {\lambda }_{{\bf{1}}}{\parallel }^{2} & = & ({\bf{y}}{T}_{z}-{\bf{y}}{\boldsymbol{{\prime} }}{T}_{z{\prime} })\,({T}_{z}{{\bf{y}}}^{T}-{T}_{z{\prime} }{{\bf{y}}{\boldsymbol{{\prime} }}}^{T})\\  & = & {\bf{y}}{T}_{z}{T}_{z}{{\bf{y}}}^{T}-2{\bf{y}}{T}_{z}{T}_{z{\prime} }{{\bf{y}}{\boldsymbol{{\prime} }}}^{T}+{\bf{y}}{\boldsymbol{{\prime} }}{T}_{z{\prime} }{T}_{z{\prime} }{{\bf{y}}{\boldsymbol{{\prime} }}}^{T}\mathrm{}.\end{array}$$

So we have

20 $$\begin{array}{rcl}\parallel {\lambda }_{{\bf{0}}}{\parallel }^{2}+\parallel {\lambda }_{{\bf{1}}}{\parallel }^{2} & = & ({\bf{y}}+{\bf{y}}{\boldsymbol{{\prime} }})\,({T}_{z}{T}_{z}+{T}_{z{\prime} }{T}_{z{\prime} })\,({{\bf{y}}}^{T}+{{\bf{y}}{\boldsymbol{{\prime} }}}^{T})\\  &  & -\,2{\bf{y}}({T}_{z}{T}_{z}+{T}_{z{\prime} }{T}_{z{\prime} }){{\bf{y}}{\boldsymbol{{\prime} }}}^{T}+2{\bf{y}}({T}_{z{\prime} }{T}_{z}-{T}_{z}{T}_{z{\prime} }){{\bf{y}}{\boldsymbol{{\prime} }}}^{T}\\  & = & 4{\rho }_{18}^{2}[({y}_{1}^{2}+{y}_{2}^{2}+{y{\prime} }_{1}^{2}+{y{\prime} }_{2}^{2})\,({z}_{1}^{2}+{z}_{2}^{2}+{z{\prime} }_{1}^{2}+{z{\prime} }_{2}^{2})\\  &  & -\,\mathrm{4(}{y{\prime} }_{1}{y}_{2}-{y}_{1}{y{\prime} }_{2})\,({z{\prime} }_{1}{z}_{2}-{z}_{1}{z{\prime} }_{2})]+{N}^{2}({y}_{3}^{2}+{y{\prime} }_{3}^{2})\,({z}_{3}^{2}+{z{\prime} }_{3}^{2}\mathrm{)}.\end{array}$$

Since ${\bf{y}},{\bf{y}}{\boldsymbol{{\prime} }},{\bf{z}},{\bf{z}}{\boldsymbol{{\prime} }}\in {{\mathbb{R}}}^{3}$ are unit vectors, we can substitute the following polar coordinate transformations into the above equation.

$$\begin{array}{ll}\{\begin{array}{rcl}{z}_{1} & = & \text{sin}\,{\alpha }_{1}\,\text{sin}\,{\alpha }_{2}\\ {z}_{2} & = & \text{sin}\,{\alpha }_{1}\,\text{cos}\,{\alpha }_{2},\\ {z}_{3} & = & \text{cos}\,{\alpha }_{1}\end{array} & \{\begin{array}{rcl}{z{\prime} }_{1} & = & \text{sin}\,{\beta }_{1}\,\text{sin}\,{\beta }_{2}\\ {z{\prime} }_{2} & = & \text{sin}\,{\beta }_{1}\,\text{cos}\,{\beta }_{2},\\ {z{\prime} }_{3} & = & \text{cos}\,{\beta }_{1}\end{array}\\ \{\begin{array}{rcl}{y}_{1} & = & \text{sin}\,{\alpha }_{3}\,\text{sin}\,{\alpha }_{4}\\ {y}_{2} & = & \text{sin}\,{\alpha }_{3}\,\text{cos}\,{\alpha }_{4},\\ {y}_{3} & = & \text{cos}\,{\alpha }_{3}\end{array} & \{\begin{array}{rcl}{y{\prime} }_{1} & = & \text{sin}\,{\beta }_{3}\,\text{sin}\,{\beta }_{4}\\ {y{\prime} }_{2} & = & \text{sin}\,{\beta }_{3}\,\text{cos}\,{\beta }_{4}.\\ {y{\prime} }_{3} & = & \text{cos}\,{\beta }_{3}\end{array}\end{array}$$

Then we have

21 $$\begin{array}{rcl}\parallel {\lambda }_{{\bf{0}}}{\parallel }^{2}+\parallel {\lambda }_{{\bf{1}}}{\parallel }^{2} & = & 4{\rho }_{18}^{2}[({\text{sin}}^{2}\,{\alpha }_{1}+{\text{sin}}^{2}\,{\beta }_{1})\,({\text{sin}}^{2}\,{\alpha }_{3}+{\text{sin}}^{2}\,{\beta }_{3})\\  &  & +\,4\,\text{sin}\,{\alpha }_{1}\,\text{sin}\,{\beta }_{1}\,\text{sin}\,{\alpha }_{3}\,\text{sin}\,{\beta }_{3}\,\text{sin}\,({\alpha }_{2}-{\beta }_{2})\,\text{sin}\,({\alpha }_{4}-{\beta }_{4})]\\  &  & +\,{N}^{2}({\text{cos}}^{2}\,{\alpha }_{1}+{\text{cos}}^{2}\,{\beta }_{1})\,({\text{cos}}^{2}\,{\alpha }_{3}+{\text{cos}}^{2}\,{\beta }_{3})\\  & = & 4{\rho }_{18}^{2}[({\text{sin}}^{2}\,{\alpha }_{1}+{\text{sin}}^{2}\,{\beta }_{1})\,({\text{sin}}^{2}\,{\alpha }_{3}+{\text{sin}}^{2}\,{\beta }_{3})\\  &  & +\,4\,\text{sin}\,{\alpha }_{1}\,\text{sin}\,{\beta }_{1}\,\text{sin}\,{\alpha }_{3}\,\text{sin}\,{\beta }_{3}\,\text{sin}\,({\alpha }_{2}-{\beta }_{2})\,\text{sin}\,({\alpha }_{4}-{\beta }_{4})]\\  &  & +\,{N}^{2}\mathrm{(2}-{\text{sin}}^{2}\,{\alpha }_{1}-{\text{sin}}^{2}\,{\beta }_{1})\,\mathrm{(2}-{\text{sin}}^{2}\,{\alpha }_{3}-{\text{sin}}^{2}\,{\beta }_{3}\mathrm{)}.\end{array}$$

Since we only need the maximum value of $\parallel {\lambda }_{{\bf{0}}}{\parallel }^{2}+\parallel {\lambda }_{{\bf{1}}}{\parallel }^{2}$ and according to the expression of $\parallel {\lambda }_{{\bf{0}}}{\parallel }^{2}+\parallel {\lambda }_{{\bf{1}}}{\parallel }^{2}$, we can assume that $0\le \text{sin}\,{\alpha }_{1},\text{sin}\,{\beta }_{1},\text{sin}\,{\alpha }_{3},\text{sin}\,{\beta }_{3}\le 1$ and $\text{sin}\,({\alpha }_{2}-{\beta }_{2})=\text{sin}\,({\alpha }_{2}-{\beta }_{2})=1$. So we get

22 $$\begin{array}{rcl}\mathop{\text{max}}\limits_{{\bf{y}},{\bf{y}}{\boldsymbol{{\prime} }},{\bf{z}},{\bf{z}}{\boldsymbol{{\prime} }}}\,\parallel {\lambda }_{{\bf{0}}}{\parallel }^{2}+\parallel {\lambda }_{{\bf{1}}}{\parallel }^{2} & = & \mathop{\text{max}}\limits_{{\alpha }_{1},{\beta }_{1},{\alpha }_{3},{\beta }_{3}}4{\rho }_{18}^{2}[({\text{sin}}^{2}\,{\alpha }_{1}+{\text{sin}}^{2}\,{\beta }_{1})\,({\text{sin}}^{2}\,{\alpha }_{3}+{\text{sin}}^{2}\,{\beta }_{3})\\  &  & +\,4\,\text{sin}\,{\alpha }_{1}\,\text{sin}\,{\beta }_{1}\,\text{sin}\,{\alpha }_{3}\,\text{sin}\,{\beta }_{3}]\\  &  & +\,{N}^{2}\mathrm{(2}-{\text{sin}}^{2}\,{\alpha }_{1}-{\text{sin}}^{2}\,{\beta }_{1})\,\mathrm{(2}-{\text{sin}}^{2}\,{\alpha }_{3}-{\text{sin}}^{2}\,{\beta }_{3})\\  & = & \mathop{\text{max}}\limits_{{\alpha }_{1},{\beta }_{1},{\alpha }_{3},{\beta }_{3}}4{\rho }_{18}^{2}\delta +{N}^{2}\delta {\prime} ,\end{array}$$

where $$\delta =({\text{sin}}^{2}\,{\alpha }_{1}+{\text{sin}}^{2}\,{\beta }_{1})\,({\text{sin}}^{2}\,{\alpha }_{3}+{\text{sin}}^{2}\,{\beta }_{3})+4\,\text{sin}\,{\alpha }_{1}\,\text{sin}\,{\beta }_{1}\,\text{sin}\,{\alpha }_{3}\,\text{sin}\,{\beta }_{3}$$ and $$\delta {\prime} =(2-{\text{sin}}^{2}\,{\alpha }_{1}-\,{\beta }_{1})$$ $$(2-{\text{sin}}^{2}\,{\alpha }_{3}-{\text{sin}}^{2}\,{\beta }_{3})$$and the maximum is over all angles of *α*_1_, *β*_1_, *α*_3_, *β*_3_ such that $$0\le \text{sin}\,{\alpha }_{1},\text{sin}\,{\beta }_{1},$$$$\text{sin}\,{\alpha }_{3},\text{sin}\,{\beta }_{3}\le 1$$. And for this maximum value we claim that it is equal to $$\text{max}\{32{\rho }_{18}^{2},4{N}^{2}\}$$.

Suppose $0\le a,\,b\le 2$ are two arbitral real numbers, then the inequality $a+b-ab\ge 0$ obviously holds. Thus we have

23 $$\begin{array}{rcl}\delta +2\delta {\prime}  & = & ({\text{sin}}^{2}\,{\alpha }_{1}+{\text{sin}}^{2}\,{\beta }_{1})\,({\text{sin}}^{2}\,{\alpha }_{3}+{\text{sin}}^{2}\,{\beta }_{3})\\  &  & +\,4\,\text{sin}\,{\alpha }_{1}\,\text{sin}\,{\beta }_{1}\,\text{sin}\,{\alpha }_{3}\,\text{sin}\,{\beta }_{3}\\  &  & +\,(2-{\text{sin}}^{2}\,{\alpha }_{1}-{\text{sin}}^{2}\,{\beta }_{1})\,(2-{\text{sin}}^{2}\,{\alpha }_{3}-{\text{sin}}^{2}\,{\beta }_{3})\\  & = & 8+3({\text{sin}}^{2}\,{\alpha }_{1}+{\text{sin}}^{2}\,{\beta }_{1})\,({\text{sin}}^{2}\,{\alpha }_{3}+{\text{sin}}^{2}\,{\beta }_{3})\\  &  & +\,4\,\text{sin}\,{\alpha }_{1}\,\text{sin}\,{\beta }_{1}\,\text{sin}\,{\alpha }_{3}\,\text{sin}\,{\beta }_{3}\\  &  & -\,4({\text{sin}}^{2}\,{\alpha }_{1}+{\text{sin}}^{2}\,{\beta }_{1}+{\text{sin}}^{2}\,{\alpha }_{3}+{\text{sin}}^{2}\,{\beta }_{3})\\  & \le  & 8.\end{array}$$

If $$32{\rho }_{18}^{2}\ge 4{N}^{2}$$, then

24 $$\begin{array}{rcl}32{\rho }_{18}^{2}-(4{\rho }_{18}^{2}\delta +{N}^{2}\delta {\prime} ) & = & 4{\rho }_{18}^{2}(8-\delta )-{N}^{2}\delta {\prime} \\  & \ge  & 8{\rho }_{18}^{2}\delta {\prime} -{N}^{2}\delta {\prime} \\  & \ge  & 0.\end{array}$$

If $$4{N}^{2}\ge 32{\rho }_{18}^{2}$$, then

25 $$\begin{array}{rcl}4{N}^{2}-(4{\rho }_{18}^{2}\delta +{N}^{2}\delta {\prime} ) & = & {N}^{2}(4-\delta {\prime} )-4{\rho }_{18}^{2}\delta \\  & \ge  & \frac{1}{2}{N}^{2}\delta -4{\rho }_{18}^{2}\delta \\  & \ge  & 0.\end{array}$$

Since

26 $$\begin{array}{rcl}\langle {\lambda }_{{\bf{0}}},{\lambda }_{{\bf{1}}}\rangle  & = & ({\bf{y}}{T}_{z{\prime} }+{\bf{y}}{\boldsymbol{{\prime} }}{T}_{z})({T}_{z}{{\bf{y}}}^{T}+{T}_{z{\prime} }{{\bf{y}}{\boldsymbol{{\prime} }}}^{T})\\  & = & 4{\rho }_{18}^{2}[({y}_{1}^{2}+{y}_{2}^{2}-{y{\prime} }_{1}^{2}-{y{\prime} }_{2}^{2})\,({z}_{1}{z{\prime} }_{1}+{z}_{2}{z{\prime} }_{2})\\  &  & +\,({z}_{1}^{2}+{z}_{2}^{2}-{z{\prime} }_{1}^{2}-{z{\prime} }_{2}^{2})\,({y}_{1}{y{\prime} }_{1}+{y}_{2}{y{\prime} }_{2})]\\  &  & +\,{N}^{2}[{z}_{3}{z{\prime} }_{3}({y}_{3}^{2}-{y{\prime} }_{3}^{2})+{y}_{3}{y{\prime} }_{3}({z}_{3}^{2}-{z{\prime} }_{3}^{2})]\\  & = & 4{\rho }_{18}^{2}[\text{sin}\,{\alpha }_{1}\,\text{sin}\,{\beta }_{1}\,\text{sin}\,({\alpha }_{2}+{\beta }_{2})\,({\text{sin}}^{2}\,{\alpha }_{3}-{\text{sin}}^{2}\,{\beta }_{3})\\  &  & +\,\text{sin}\,{\alpha }_{3}\,\text{sin}\,{\beta }_{3}\,\text{sin}\,({\alpha }_{4}+{\beta }_{4})\,({\text{sin}}^{2}\,{\alpha }_{1}-{\text{sin}}^{2}\,{\beta }_{1})]\\  &  & +\,{N}^{2}[\text{cos}\,{\alpha }_{1}\,\text{cos}\,{\beta }_{1}({\text{cos}}^{2}\,{\alpha }_{3}-{\text{cos}}^{2}\,{\beta }_{3})\\  &  & +\,\text{cos}\,{\alpha }_{3}\,\text{cos}\,{\beta }_{3}({\text{cos}}^{2}\,{\alpha }_{1}-{\text{cos}}^{2}\,{\beta }_{1})],\end{array}$$

we see that the maximum value of $\mathop{\text{max}}\limits_{{\bf{y}},{\bf{y}}{\boldsymbol{{\prime} }},{\bf{z}},{\bf{z}}{\boldsymbol{{\prime} }}}\,\parallel {\lambda }_{{\bf{0}}}{\parallel }^{2}+\parallel {\lambda }_{{\bf{1}}}{\parallel }^{2}$ can be obtained for ${\lambda }_{{\bf{0}}}$ and ${\lambda }_{{\bf{1}}}$ with ${\lambda }_{{\bf{0}}}\perp {\lambda }_{{\bf{1}}}$. Then by lemma 1 we have

27 $$S(\rho )=\text{max}\{8\sqrt{2}|{\rho }_{18}|,4|N|\},$$

where $\rho$ stands for ${\rho }_{ABC}(t)$ or ${\rho }_{Abc}(t)$. When $\theta =\pi /4$ and $t=0$, the state ${\rho }_{ABC}(t)$ is exactly the GHZ state $|GHZ\rangle =(|eee\rangle +|ggg\rangle )/\sqrt{2}$. Under these conditions, we have $S({\rho }_{ABC}(0))=4\sqrt{2}$, which is coincide with the previous results.

Similarly we have

28 $$S(\rho )=\text{max}\{8\sqrt{-\,2{\rho }_{18}^{2}},4|N|\},$$

where $\rho$ stands for ${\rho }_{abc}(t)$ or ${\rho }_{ABc}(t)$.

**Figure 1:**
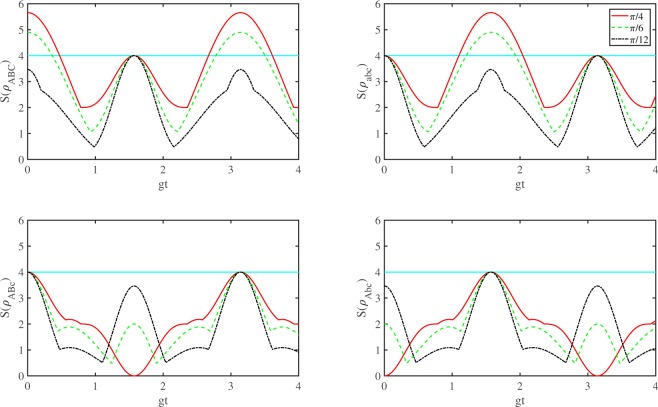
Plots of $S({\rho }_{ABC}),S({\rho }_{abc}),S({\rho }_{ABc})$ and $S({\rho }_{Abc})$ as a function of *gt* for the case of ${g}_{A}={g}_{B}={g}_{C}=g$.

In Fig. 1, we plot *S*(*ρ*_*ABC*_), *S*(*ρ*_*abc*_), *S*(*ρ*_*ABc*_) and *S*(*ρ*_*Abc*_) as a function of $$gt$$ for the case of $${g}_{A}={g}_{B}={g}_{C}=g$$ and $$\theta =\pi /4,\pi /6,\pi /12$$. As we can see from the picture, there exist some time intervals in which $${\rho }_{abc}$$ and $${\rho }_{abc}$$ violate the Svetlichny inequality for $$\theta =\pi \mathrm{/4,}\pi \mathrm{/6}$$. And in other time intervals, the corresponding subsystems all satisfies the Svetlichny inequality as the time evolutions. After a simple calculation by (27) and (28), if $$\theta =\pi /4$$ and $$gt$$ is located in the intervals $$[0,0.4751)$$, $$(2.6701+k\pi ,3.6131+k\pi )$$, where $$k=0,1,2\cdots $$, then the state of the subsystem *ABC* is known for sure to be genuinely nonlocal. If $$\theta =\pi /6$$, then the intervals of $$gt$$ such that the state is genuinely nonlocal are $$[0,0.3635)$$, $$(2.7781+k\pi ,3.5051+k\pi )$$, where $$k=0,1,2\cdots $$. Similarly for the subsystem $$abc$$ we obtain the intervals $$(1.0993+k\pi ,2.0423+k\pi )$$ and $$(1.2073+k\pi ,1.9343+k\pi )$$, where $$k=0,1,2\cdots $$, for the value of $$\theta =\pi /4$$ and $$\theta =\pi /6$$, respectively. As for the other two subsystems $$ABc$$ and $$Abc$$, the maximal value of $$S({\rho }_{abc})$$ or $$S({\rho }_{Abc})$$ is 4 calculated by our expression (27) and (28) as the time evolutions. We demonstrate $$S({\rho }_{ABc})\le 4$$ for $$\theta =\pi /4$$ and the other cases can be derived in the similar way. Since $$8\sqrt{-\,2{\rho }_{18}^{2}}=4\sqrt{2}|\text{sin}\,(gt)-{\text{sin}}^{3}\,(gt)|\le \frac{8\sqrt{6}}{9} < 4$$ and $$4|N|=2|1+{\text{cos}}^{3}\,(2gt)|\le 4$$, we have $$S({\rho }_{ABc})=\text{max}\{8\sqrt{-\,2{\rho }_{18}^{2}},4|N|\}\le 4$$. In ref. ^14^ the authors find that the three-tangle magnitudes of the subsystem ABc and Abc are smaller than those of the subsystem ABC and abc. When we investigate the genuine tripartite nonlocalities of these subsystems, maybe the correlations of the $$(Abc)$$ and $$(Abc)$$ systems are not able to be tested by the Svetlichny inequalities. From a physical point of view, we don’t know very well this peculiar phenomenon that happened among these subsystems. This results may implicit that the correlation among three atoms or among three cavities is more intense than the subsystems of atoms and cavities mixed together.

**Figure 2:**
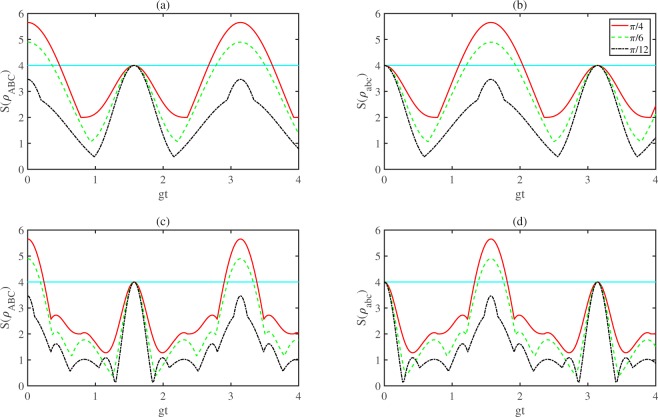
Plots of $S({\rho }_{ABC})$ and $S({\rho }_{abc})$ as a function of *gt*, where ${g}_{A}={g}_{B}={g}_{C}=g$ for (**a**,**b**) and ${g}_{A}={g}_{B}=\frac{1}{3}{g}_{C}=g$ for (**c**,**d**).

In Fig. 2, we plot $S({\rho }_{ABC})$ and $S({\rho }_{abc})$ for $|{\Phi }_{ABC}\rangle$ as a function of $gt$ for both the cases of equal (Fig. 2(a,b)) and different (Fig. 2(c,d) with ${g}_{A}={g}_{B}={g}_{C}/3=g$) coupling constants. We next analyze the genuine nonlocality of the corresponding atoms and cavities and obtain that the nonlocality losses (gains) of atomic subsystems while the nonlocality gains (losses) of cavities. Set $S({\rho }_{ABC})=4$, after a simple calculate by expression (27), we see that the genuine tripartite nonlocality changes in the time interval $T$ which is defined as $T\,:0\le {g}_{\text{max}}t\le \frac{\pi }{2}$, where ${g}_{\text{max}}=\text{max}\{{g}_{A},{g}_{B},{g}_{C}\}$. Formally, one can treat the cavity fields during $T$ as a dissipation factor for the atoms and map a dissipative evolution (refs. ^13,20,21^). This dissipative evolution can be looked upon as a decaying process obeyed by an exponential rule exp$(\,-\,\gamma t{\prime} )$; onto the JC dymamics by identifying between the *t* and *t*′ as exp$(\,-\,\gamma t{\prime} )={\text{cos}}^{2}\,({g}_{\text{max}}t)$^22^. Then, $t{\prime} \to \infty$ is corresponding to ${g}_{\text{max}}t\to \frac{\pi }{2}$. Comparing Fig. 2(a–d)), we find that the atoms (cavities) are genuine tripartite nonlocality for some time interval before $gt=\frac{\pi }{2}$, no matter whether the coupling constants are equal or different. Comparing Fig. 2(a–d)), roughly speaking, the genuine tripartite nonlocality of atoms decreases while the nonlocality of cavities increases and vice versa.

### Genuine nonlocality of the |Ψ_*ABC*_〉 type GHZ-like state

In this section, we choose the GHZ-like state $|{\Psi }_{ABC}\rangle$ in (3) as an atomic initial state, thus the initial state of the total system is

29 $$\begin{array}{rcl}|\Psi (0)\rangle  & = & |{\Psi }_{ABC}\rangle \otimes |000{\rangle }_{abc}\\  & = & {(\text{cos}\theta |eeg\rangle }_{ABC}+\text{sin}\,\theta |gge{\rangle }_{ABC})\otimes |000{\rangle }_{abc}.\end{array}$$

At time $t$, the state of the system in Eq. (29) evolves into

30 $$\begin{array}{rcl}|\Psi (t)\rangle  & = & {C}_{1}|eeg000\rangle +{C}_{2}|egg010\rangle +{C}_{3}|geg100\rangle +{C}_{4}|ggg110\rangle \\  &  & +\,{C}_{5}|gge110\rangle +{C}_{6}|ggg001\rangle ,\end{array}$$

where the six coefficients ${C}_{i}(i=1\ldots 6)$ are not written here since they are the same as Eq. (18) in ref. ^13^ except the phase factors do not affect the final results.

As the atoms $A$ and $B$ in the GHZ-like state (3) are permutationally invariant, the whole highly symmetry six-qubit system only has six inequivalent non-interaction subsystems $ABC$, $abc$, $ABc$, $Abc$, $ACb$ and $Cab$. The nonzero entities of the density matrices of the six subsystems can be found in refs. ^13,14^ and we also leave these data out here.

As for the calculation of $S(\rho )$, it is similarly to the last section. We only write the results down here

31 $$S(\rho )=\text{max}\{8\sqrt{2}|{\rho }_{27}|,4|N{\prime} |\},$$

where $\rho$ stands for ${\rho }_{ABC}(t)$ or ${\rho }_{Abc}(t)$ and $N{\prime} ={\rho }_{11}-{\rho }_{22}-{\rho }_{33}-{\rho }_{55}+{\rho }_{77}$.

And

32 $$S(\rho )=\text{max}\{8\sqrt{-\,2{\rho }_{27}^{2}},4|N{\prime} |\},$$

where $\rho$ stands for ${\rho }_{abc}(t)$ or ${\rho }_{ABc}(t)$ and $N{\prime} ={\rho }_{11}-{\rho }_{22}-{\rho }_{33}-{\rho }_{55}+{\rho }_{77}$.

For the other two subsystems $ACb$ and $Cab$ we have

33 $$S({\rho }_{ACb})=\text{max}\{8\sqrt{-\,2{\rho }_{36}^{2}},4|{N}_{1{\prime} }|\},\,S({\rho }_{Cab})=\text{max}\{8\sqrt{2}|{\rho }_{45}|,4|{N}_{2{\prime} }|\},$$

where $${N{\prime} }_{1}={\rho }_{11}-{\rho }_{22}-{\rho }_{33}-{\rho }_{55}+{\rho }_{66}$$ and $${N{\prime} }_{2}={\rho }_{11}-{\rho }_{22}-{\rho }_{33}+{\rho }_{44}-{\rho }_{55}$$.

**Figure 3:**
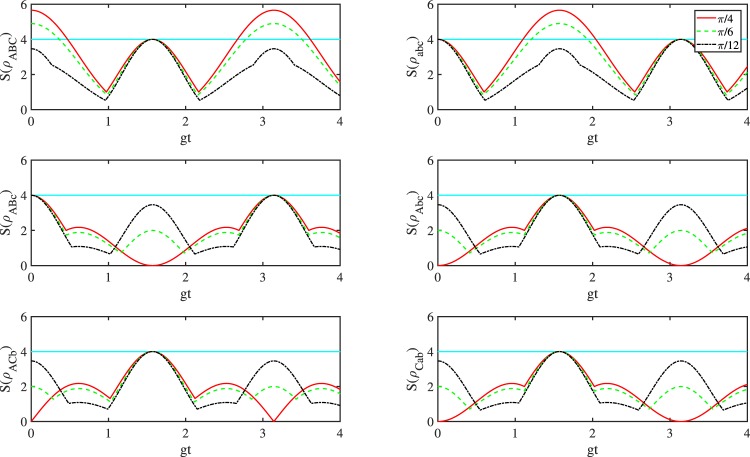
Plots of $S({\rho }_{ABC}),S({\rho }_{abc}),S({\rho }_{ABc})$, $S({\rho }_{Abc})$, $S({\rho }_{ACb})$ and $S({\rho }_{Cab})$ as a function of *gt* for the case of ${g}_{A}={g}_{B}={g}_{C}=g$.

In Fig. 3, we plot $S({\rho }_{ABC}),S({\rho }_{abc}),S({\rho }_{ABc})$, $S({\rho }_{Abc})$, $S({\rho }_{ACb})$ and $S({\rho }_{Cab})$ as a function of $gt$ for the case of ${g}_{A}={g}_{B}={g}_{C}=g$ and $\theta =\pi /4,\pi /6,\pi /12$. In the case of GHZ-like state $|{\Psi }_{ABC}\rangle$ we have a similar picture to the former case. As we can see from the picture, there exist some time intervals in which ${\rho }_{ABC}$ and ${\rho }_{abc}$ violate the Svetlichny inequality for $\theta =\pi /4,\pi /6$. These time intervals are also periodic similar to the GHZ-like state $|{\Phi }_{ABC}\rangle$. While the exact intervals such that the corresponding state is genuinely nonlocal is coincide with the situation of GHZ-like state $|{\Phi }_{ABC}\rangle$ as initial state, since we choose the same coupling constants. And in other cases the states all satisfy the Svetlichny inequality as the time evolutions.

**Figure 4:**
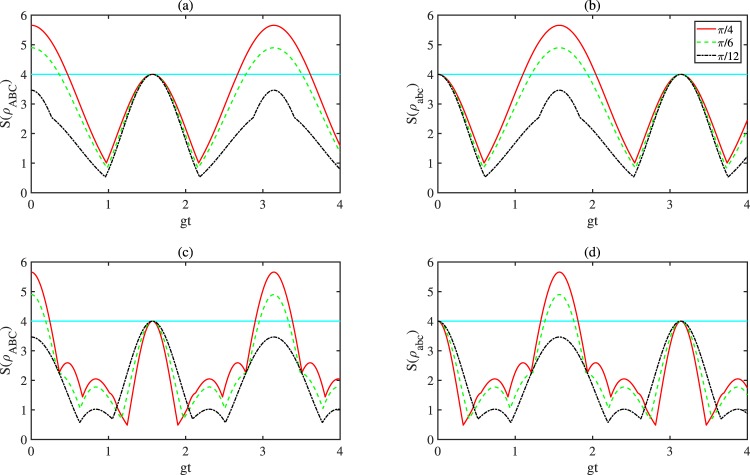
Plots of $S({\rho }_{ABC})$ and $S({\rho }_{abc})$ as a function of *gt* for the case of ${g}_{A}={g}_{B}=\frac{1}{3}{g}_{C}=g$.

In Fig. 4, we plot $S({\rho }_{ABC})$ and $S({\rho }_{abc})$ for $|{\Psi }_{ABC}\rangle$ as a function of $gt$ for both the cases of equal (Fig. 4(a,b)) and different (Fig. 4(c,d) with ${g}_{A}={g}_{B}={g}_{C}/3=g$) coupling constants. Easily, we find that Fig. 4 is similar with Fig. 2 for the chosen coupling constants. Therefore, we get the corresponding analysis for Fig. 4 as before in the dissipation language.

## Discussion

We investigate the genuine nonlocality dynamics in a triple Jaynes-Cummings model with the two types of GHZ-like states as initial states based on the violation of the Svetlichny inequality. We calculate and get the exact analytical expressions for all inequivalent non-interaction subsystems. For the two types of GHZ-like states as initial states, we know there are certain time intervals and angles $\theta$ that the corresponding states violate the Svetlichny inequality for the subsystems $ABC$ and $abc$. This means the corresponding subsystems are genuine tripartite nonlocality. Since the quantum state of three atoms and of three cavities all do not display nonlocality via the MABK inequality as shown in ref. ^15^, it seems that MABK inequality is not always the optimum way to test the local realism according to our results. As for the other cases, we know the Svetlichny inequalities of the corresponding subsystems hold for all possible Svetlichny operators. Since $S(\rho ) > 4$ is a sufficient condition for the three-qubit state $\rho$ being genuinely nonlocal, we don’t know whether the subsystems are genuinely nonlocal or not for the cases which the Svetlichny inequalities hold. Physically speaking, the subsystem of three atoms or three cavities may be more intense correlation than the other subsystems consist of atoms and cavities.

## Methods

For any three-qubit state $\rho$, the genuine tripartite nonlocality $S(\rho )$ can be calculated by Theorem 1 in ref. ^16^

34 $$S(\rho )=2\sqrt{F({T}_{1},{T}_{2},{T}_{3})},$$

where

$$F({T}_{1},{T}_{2},{T}_{3})=\mathop{\text{max}}\limits_{{\bf{y}},{\bf{y}}{\boldsymbol{{\prime} }},{\bf{z}},{\bf{z}}{\boldsymbol{{\prime} }}}\,\frac{1}{2}[\parallel {\lambda }_{{\bf{0}}}{\parallel }^{2}+\parallel {\lambda }_{{\bf{1}}}{\parallel }^{2}+\sqrt{{(\parallel {\lambda }_{{\bf{0}}}{\parallel }^{2}+\parallel {\lambda }_{{\bf{1}}}{\parallel }^{2})}^{2}-4{\langle {\lambda }_{{\bf{0}}},{\lambda }_{{\bf{1}}}\rangle }^{2}}]$$

and the maximum is taken over all possible measurement variables **y**, **y**′, **z** and **z**′. If we detect that $S(\rho ) > 4$, then we know the corresponding state $\rho$ is genuinely nonlocal by the violation of Svetlichny inequality.
